# Sperm YOLOv8E-TrackEVD: A Novel Approach for Sperm Detection and Tracking

**DOI:** 10.3390/s24113493

**Published:** 2024-05-28

**Authors:** Chongming Zhang, Yaxuan Zhang, Zhanyuan Chang, Chuanjiang Li

**Affiliations:** College of Information, Mechanical and Electrical Engineering, Shanghai Normal University, Shanghai 200234, China; czhang@shnu.edu.cn (C.Z.); 1000528175@smail.shnu.edu.cn (Y.Z.); licj@shnu.edu.cn (C.L.)

**Keywords:** male infertility, sperm detection, YOLOv8 enhancement, DeepOCSORT, automatic semen analysis

## Abstract

Male infertility is a global health issue, with 40–50% attributed to sperm abnormalities. The subjectivity and irreproducibility of existing detection methods pose challenges to sperm assessment, making the design of automated semen analysis algorithms crucial for enhancing the reliability of sperm evaluations. This paper proposes a comprehensive sperm tracking algorithm (Sperm YOLOv8E-TrackEVD) that combines an enhanced YOLOv8 small object detection algorithm (SpermYOLOv8-E) with an improved DeepOCSORT tracking algorithm (SpermTrack-EVD) to detect human sperm in a microscopic field of view and track healthy sperm in a sample in a short period effectively. Firstly, we trained the improved YOLOv8 model on the VISEM-Tracking dataset for accurate sperm detection. To enhance the detection of small sperm objects, we introduced an attention mechanism, added a small object detection layer, and integrated the SPDConv and Detect_DyHead modules. Furthermore, we used a new distance metric method and chose IoU loss calculation. Ultimately, we achieved a 1.3% increase in precision, a 1.4% increase in recall rate, and a 2.0% improvement in mAP@0.5:0.95. We applied SpermYOLOv8-E combined with SpermTrack-EVD for sperm tracking. On the VISEM-Tracking dataset, we achieved 74.303% HOTA and 71.167% MOTA. These results show the effectiveness of the designed Sperm YOLOv8E-TrackEVD approach in sperm tracking scenarios.

## 1. Introduction

Infertility affects approximately 8–12% of couples globally, encompassing both psychological and physiological aspects [1]. Male infertility factors contribute to about 40–50% of all documented cases. Given these statistics, male infertility has garnered attention from researchers and medical practitioners, particularly over the past half-century [2].

Male infertility issues may be linked to poor sperm viability (oligospermia), low sperm production (oligozoospermia), or morphological abnormalities (teratozoospermia) [3]. A combination of one or more of these issues is utilized to categorize different forms of male-factor infertility. Currently, there is a lack of standardized diagnostic methods, leading to the prescription of treatments based on experience, which often remains questionable.

Semen analysis aids in determining sperm count or functional impairments leading to infertility. The World Health Organization (WHO) highlights sperm motility as one of the most critical attributes for assessing semen quality. Infertility is considered a global health issue affecting many. In semen examination, tracking and selecting healthy sperm presents a challenging task.

Severe male factor cases necessitate the use of Intracytoplasmic Sperm Injection (ICSI). During ICSI, embryologists evaluate sperm motility and morphology to decide on sperm usage for fertilization. The WHO Laboratory Manual [4] and Auger [5] both report characteristics of normal morphology sperm. In clinical practice, embryologists use these standards based on experience, making morphological assessments in ICSI personal, subjective, and non-reproducible [6].

Moreover, the success of manual sperm drawing is dependent on personnel, often lacking repeatability due to personnel changes or inadequate training. Designing an automated semen analysis system that offers sufficient consistency intra- and inter-laboratory at an affordable cost is crucial.

Another method for assessing male fertility involves the use of Computer-Assisted Sperm Analysis (CASA) [7] with various staining procedures. Comprising specific software, high-resolution cameras, and microscopes, this system standardizes sperm examination. However, limitations such as high costs, stringent requirements for algorithm parameter definitions, and questionable accuracy have restricted CASA systems’ laboratory use. Additionally, sperm staining might compromise sperm vitality and integrity [8]. Chemical dyes could affect sperm DNA integrity or other crucial biological properties, increasing the complexity of the ICSI procedure. Stained sperm may alter appearance, complicating the selection process vital for choosing healthy and active sperm in ICSI.

Given the shortcomings of manual solutions and potential issues with sperm staining, automated technology is crucial for analyzing human sperm morphology [9]. Hence, designing efficient, accurate, non-staining sperm analysis and classification algorithms to select optimal sperm before ICSI is both a challenging and trending task. In other words, an automated method for selecting the best sperm without staining during the ICSI process would be more ideal for embryologists, potentially increasing fertilization and pregnancy rates. Compared to CASA systems and visual assessment methods, employing machine and deep learning approaches for automated sperm morphology analysis offers a cost-effective and objective solution, representing an attractive and promising alternative.

This paper introduces an approach that combines an enhanced YOLOv8 algorithm for detecting small targets with an improved DeepOCSORT tracking algorithm, specifically targeting the sperm detection environment and offering higher accuracy and stability in the tracking phase that follows detection. The contributions of this paper are summarized as follows:

1. Based on the YOLOv8 network model, we have made targeted improvements for sperm detection by integrating an enhanced attention mechanism and techniques specifically designed for detecting small objects.
2. Algorithms for tracking small-scale objects like sperm, improving the precision and reliability of sperm tracking for fertility analysis and related applications.

Through these innovations, the proposed method addresses the limitations of existing MOT algorithms in tracking small-scale objects like sperm, improving the precision and reliability of sperm tracking for fertility analysis and related applications.

## 2. Related Works

Machine learning algorithms hold significant importance in sperm morphology classification. Jia Qian [10] and others have employed principal component analysis and scale-invariant feature transform to extract features from sperm images, integrating K-nearest neighbors (KNN) and backpropagation neural network (BPNN) for classification. Structure-aware multi-object discovery [11] uses gradient-oriented histograms (HOG) for feature extraction, and support vector machine classifiers for object detection can effectively reduce lighting interference and capture multi-directional edge information using the Minimum Spanning Tree (MST) method to construct structural relationships between objects and real-time updates of structural constraints through motion measurement to maintain spatial consistency of multiple targets. Shaker et al. [12] introduced a novel feature extractor for delineating sperm head contours through elliptical features. Combining these features with a linear discriminant analysis (LDA) classifier significantly improved the distinction among normal, tapered, pyriform, and amorphous sperm categories. While traditional machine learning algorithms play a crucial role in semen classification, their reliance on manually crafted features for input into classifiers is notably inconvenient [13].

In recent years, deep learning algorithms have been utilized for analyzing sperm morphology and detecting sperm, requiring only image input without reliance on manual processing. An automated system leveraging deep learning technologies employed pre-trained convolutional neural networks (CNNs), including GoogleNet and VGG16, to identify sperm abnormalities. By conducting a weighted majority vote on CNN predictions, classification performance was significantly enhanced, achieving accuracy rates of 94% and 73.2% on the Human Sperm Head Morphology dataset (HuSHeM) and Sperm Morphology Image dataset (SCIAN-Morpho), respectively [14]. Mahali and colleagues [15] introduced a deep learning architecture utilizing the capabilities of Swin Transformer and MobileNetV3. The SwinMobile model effectively extracts essential features from sperm images and reduces noise, improving the accuracy of sperm morphology classification. The accuracy rates achieved were 95.4% on the Sperm Videos and Images Analysis dataset (SVIA), 97.6% on HuSHem, and 91.7% on SMIDS. However, these approaches merely apply existing deep neural networks to the context of sperm detection and do not specifically tailor network modifications to address the unique challenges present in sperm detection [16]. Traditional target detection algorithms, such as CNNs, are typically more effective at extracting features of larger targets but may lack sensitivity to smaller objects, which require more precise localization to differentiate between closely situated targets.

Sperm vitality is also among the most crucial attributes for evaluating semen quality. Zhu and colleagues proposed a method that combines graph theory and optical flow techniques to effectively track sperm in microscopic videos [17]. Haugen et al. [18] utilized deep convolutional neural networks (DCNN) and Lucas–Kanade optical flow estimation to automatically classify sperm into different motility categories recommended by the WHO through the analysis of 65 video samples. They simplified the tracking process from one frame to the next by refining weight conditions and quantifying trust flow, effectively addressing the issue of match failures between adjacent frames. Valiuškaitė et al. [19] assessed sperm vitality by precisely segmenting and tracking the central coordinates of sperm heads and calculating their movement speed. Their research achieved a 91.77% accuracy rate in sperm head detection on the VISEM sperm sample video dataset, demonstrating a high correlation (Pearson’s r = 0.969) with laboratory analysis methods. Zheng et al. [20] used Gaussian mixture models to identify moving sperm in videos, Kalman filtering to predict their future positions, and the Hungarian algorithm to match predicted positions with actual detected ones, ensuring accurate tracking of each sperm’s trajectory. These methods may face computational challenges when dealing with a large number of sperm and depend on the quality of images and frame rates, often underperforming with low-quality video data.

The aforementioned traditional tracking methods, in comparison to detection-based multi-object tracking (MOT) algorithms, exhibit notable limitations in terms of environmental adaptability, target identification and classification, handling complex scenes, and long-term tracking. Detection-based multi-object tracking has become the most popular paradigm within the MOT domain, dividing the problem into two subtasks. The first task involves detecting targets within each frame, and the second task consists of associating these targets across different frames. The association task is primarily solved by explicitly or implicitly leveraging strong cues, including spatial and appearance information. Takuma, Sato [21], and colleagues employed YOLOv3 for object detection, followed by the SORT algorithm for object tracking. This approach allows for simultaneous morphological assessment and tracking, offering rapid evaluation speeds. Studies indicate that this model achieves high accuracy in detecting abnormal sperm and exhibits excellent performance in tracking sperm, with most objects being tracked comprehensively. However, the model may misidentify unclear sperm, and its accuracy is limited by the movement of sperm under the microscope and the depth of focus. The multi-object tracking (MOT) algorithms discussed previously do not take into consideration re-identification (ReID) features based on appearance, focusing solely on the distance loss between two feature vectors in their cost matrix, which results in diminished tracking accuracy.

## 3. Methodology

**Figure 1:**
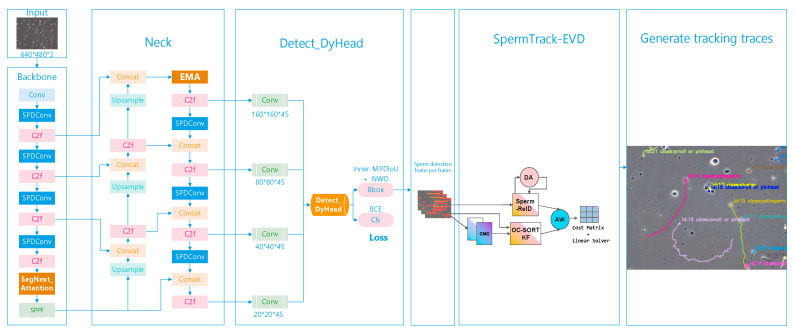
The architecture of Sperm YOLOv8E-TrackEVD.

The sperm multi-object tracking algorithm proposed in this paper is primarily composed of two parts: (1) an enhanced YoloV8 network model that has been specifically improved to enhance the detection capability for multiple small sperm targets (SpermYOLOv8-E); (2) an improved DeepOCSORT tracking algorithm that features modifications to the visual appearance module for accurate and stable tracking of sperm movement trajectories (SpermTrack-EVD). The overall structure of the algorithm is shown in Figure 1.

### 3.1. SpermYOLOv8-E

The YOLOv8, an advanced object detection model, integrates several innovative techniques to enhance detection precision and speed while maintaining model efficiency. Despite innovations and optimizations across various aspects to enhance detection performance and efficiency, the standard YOLOv8 algorithm still faces application shortcomings in environments with multiple small targets. These deficiencies primarily stem from the universality of its design and optimization, potentially leading to underperformance in specific scenarios.

Semen video datasets typically feature a resolution of 640 × 480 pixels, with normal sperm sizes ranging from 5 to 7 pixels. To effectively detect densely packed small objects like sperm, it is necessary to find the optimal balance in receptive field size that captures sufficient context without neglecting target details. Our study initially set the receptive field size to 9 pixels based on the average size of sperm. The World Health Organization’s guidelines for sperm analysis, as detailed in its Laboratory Manual [22], recommend analyzing motion trajectories for at least 200 active sperm per sample to accurately capture sperm motility parameters. This underscores the need for algorithms capable of detecting multiple small targets within such environments.

Detecting multiple small targets poses significant challenges due to their low resolution and limited features, complicating accurate localization and recognition, especially amid environmental noise. The small area covered by these targets in images makes bounding box localization difficult, where minor predictive errors can greatly affect accuracy. This necessitates a smaller receptive field to capture the details of each sperm. However, the dense distribution of sperm also demands a sufficiently large receptive field to include enough contextual information for differentiating adjacent sperm. Small targets often cluster, leading to issues in distinguishability post-downsampling and challenges in bounding box regression due to non-maximum suppression and close proximity, impacting model training and convergence. Additionally, the lack of datasets with adequate small target samples hinders performance in small target detection. These challenges underscore the need for tailored techniques and strategies in the detection of multiple small targets.

**Figure 2:**
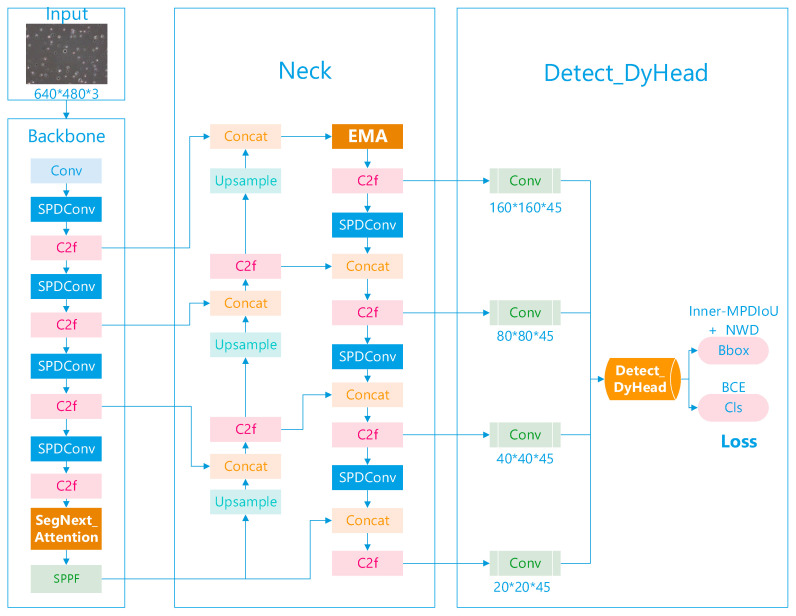
The architecture of SpermYOLOv8-E.

To further enhance the performance of YOLOv8, this study introduces an enhanced YOLOv8 algorithm that incorporates attention mechanisms to emphasize key features of sperm, improving the recognition of low-resolution small targets. This approach utilizes contextual information to address issues of sperm clustering and overlap; it adds a small target detection layer for multi-scale feature extraction, enhancing localization accuracy. Additionally, the inclusion of the SPDConv module retains fine-grained information on small targets during the downsampling process, reducing loss. The use of a new distance metric and IoU loss calculation method improves the feature extraction process. The proposed network architecture is illustrated in Figure 2.

Add a small target detection layer. YOLOv8 employs a larger downsampling factor, gradually reducing the dimension of the feature maps as it processes the input image in order to extract higher-level semantic information. This design is highly effective for detecting large-scale objects, as they retain prominent features on the higher layer feature maps. However, for small objects such as sperm, the visual information in the sperm images quickly diminishes with the reduction of the feature maps, making it challenging for the network to learn effective information about sperm images from the deeper feature maps.

To address this issue, this paper proposes the strategy of incorporating small target detection layers (STDL) into the object detection network. The core idea of this method is to utilize the feature maps from the shallower layers of the network, as these layers retain a significant amount of the original image’s detail information, such as the edges, tails, color, and head vacuole details of the sperm, which are crucial for the recognition of sperm. These shallower feature maps are then concatenated or fused with the deeper feature maps, combining the shallow layer’s detail information with the deep layer’s semantic information, thereby enhancing the network’s ability to detect sperm. This enhancement boosts the model’s capability to detect very small and densely packed targets, enhancing sensitivity and precision in locating adjacent or overlapping sperm cells.

**Figure 3:**
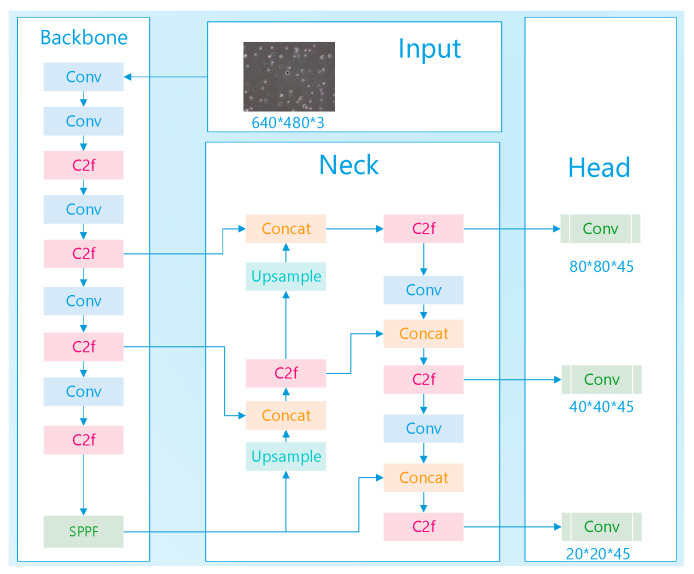
Original Structure.

**Figure 4:**
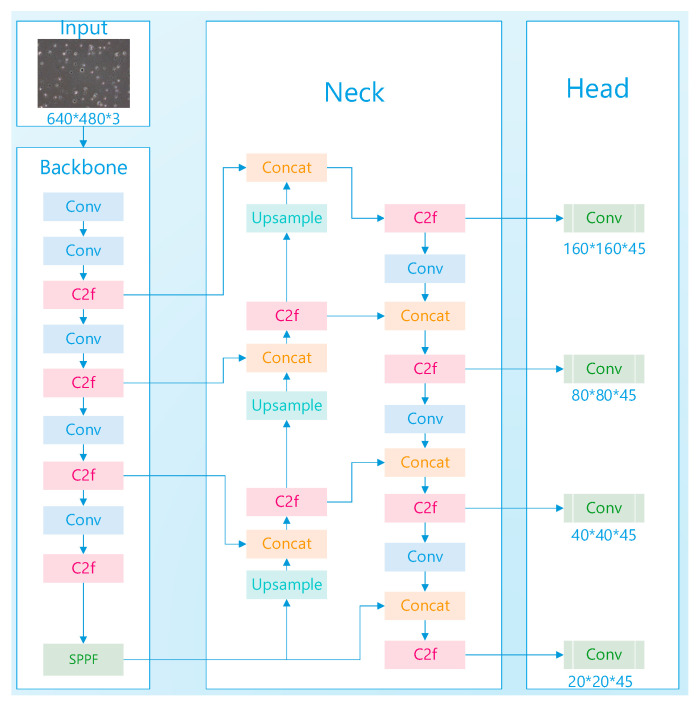
Structure After Adding a Small Object Detection Layer.

In the original architecture, three detection heads were deployed, targeting objects of different sizes: an 80 × 80 detection head for small objects, a 40 × 40 detection head for medium-sized objects, and a 20 × 20 detection head for large objects, as shown in Figure 3. To enhance detection performance for very small objects, it is proposed to add a higher resolution 160 × 160 detection layer above the 80 × 80 layer. To introduce the 160 × 160 detection head, the 160 × 160 feature map from the backbone network must be fused with the corresponding feature map in the neck. Given that the original neck design does not contain a 160 × 160 feature map, an upsampling operation is performed on the 80 × 80 feature map to achieve the desired resolution. The 160 × 160 feature map obtained after upsampling in the neck is then concatenated with the 160 × 160 feature map from the backbone network. This fusion operation facilitates the addition of a dedicated detection layer for very small objects at the 160 × 160 scale, which is then input into the detection head, as illustrated in Figure 4. This modification aims to improve the network’s sensitivity and accuracy in detecting very small objects by leveraging the detailed spatial information available at higher resolutions and the semantic information encoded by the deeper layers of the network.

Introducing an Attention Mechanism. As a deep convolutional network, YOLOv8 fundamentally relies on convolution operations to extract image features, with the 3 × 3 convolution kernel being one of the most commonly used configurations. Such convolution operations possess a strong locality, meaning the network considers only a small local area of the image at each operation, potentially overlooking the global contextual information within the image. This over-reliance on local features may lead to incomplete feature extraction, as it fails to effectively recognize and enhance features related to object detection, especially in environments with cluttered backgrounds or when the objects are small. The lack of a mechanism to focus on key areas cannot effectively extract distinctive features from small regions, affecting the accurate identification and localization of small objects.

To address the excessive locality and insufficient globality of the conventional YOLOv8 model, making it unsuitable for sperm detection environments, this paper introduces two different attention mechanisms aimed at capturing global contextual information, increasing the model’s receptive field size and enhancing the network’s focus on sperm features. It is specifically designed to detect small objects such as sperm, making the model focus on the most relevant features of sperm cells. Even if sperm overlap or adhere, it can improve detection accuracy. Meanwhile, these mechanisms increase the receptive field and improve feature extraction efficiency, helping the model better understand the distribution and interactions of sperm within the image.

In earlier applications of attention mechanisms, they were often applied at the primary levels of the network to enhance the focus on global information of the image and reduce unnecessary shallow feature information from the background [23]. However, this approach might lead to issues such as overgeneralization and information loss at the initial stages. The primary layers (such as the first few layers of a convolutional network) typically extract low-level features, which are quite general and not significantly different for various tasks. Over-focusing on these features may distract the model from areas contributing to the final classification task, leading to wasted computational resources. Attention mechanisms are more often introduced into middle and higher levels of the network [24,25,26], yet in higher levels, there might be issues of overfitting and loss of spatial contextual representation.

To overcome these issues, this paper proposes applying the attention mechanism between the middle and high layers, achieving a better balance of focus on both local and global information. The middle layers typically have better abstract representation capabilities, reducing the risk of overgeneralization and information loss while retaining important information. Furthermore, by carefully designing the placement of attention modules, the most suitable locations can be selected based on the depth and complexity of the network’s feature representations to maximize the network’s focus on sperm features.

**Figure 5:**
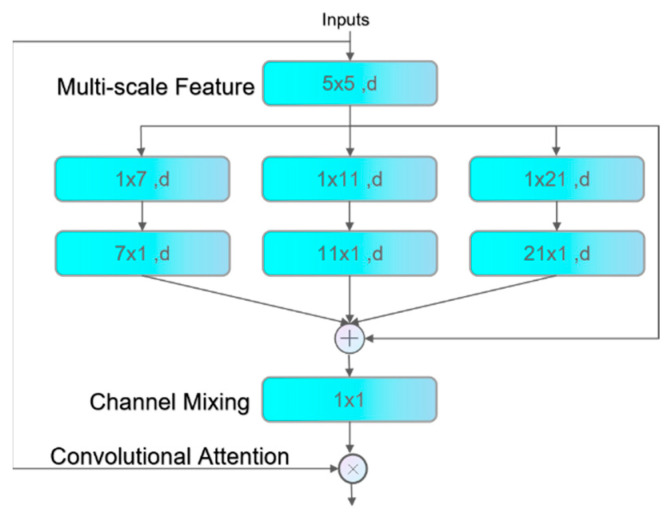
MSCA.

SegNext_Attention. SegNext_Attention [27] is a multi-scale convolutional attention (MSCA) module designed to enhance the model’s understanding of images by aggregating information across different scales, as illustrated in Figure 5.

The module comprises three parts. Initially, the module utilizes depth-wise convolution to aggregate local information. This is an efficient convolutional method capable of capturing small area details in the input while reducing parameter and computational costs, a crucial step for the model to enhance its understanding of image granularity. Subsequently, multi-branch depth-wise dilated convolutions capture multi-scale context. These specific convolutions can process areas of varying sizes, aiding the model in understanding features across various scales. Finally, 1 × 1 convolutions are employed to model the relationships between different channels, with their output directly used as attention weights to reweight the inputs of the MSCA module. This step allows the model to adjust the importance of different feature channels, further optimizing the focus on critical information.

Overall, the MSCA module, through the collaborative effort of these three core components, significantly improves the depth learning model’s depth and accuracy of image content understanding.

The MSCA (multi-scale convolutional attention) module’s formula is as follows, where DWConv denotes depth-wise convolution.

(1) $$\mathrm{Att}=Conv_{1\times 1}(\overset{3}{\underset{i=0}{\sum }}Scale_{i}(\mathrm{DW}Conv(F))).$$

(2) Out=Att⊗F.

Here, F represents the feature input entering the module, while Att and Out denote the attention map and the final output, respectively. The operation ⊗ signifies element-wise matrix multiplication. DW stands for depth-wise convolution, and Scale_i_ ∈ {0,1,2,3} denotes the scale factor of the ith branch in Figure 5. Scale_0_ is the shortcut branch, which aids in maintaining the flow of information during network training, mitigating the vanishing gradient problem, and allowing the network to learn the identity mapping of the input data. As described in the literature [28], two depth-wise dilated convolutions are deployed within each branch to approximate the effect of standard depth convolutions with large kernels. The kernel sizes for these branches are set to 7, 11, and 21, respectively. The adoption of depth-wise dilated convolutions is based on two considerations. Firstly, the dilated convolution design is lightweight, significantly reducing the parameter count and computational complexity; for instance, to simulate a standard 7 × 7 two-dimensional convolution kernel, only a set of 7 × 1 and 1 × 7 convolution operations are required. Secondly, dilated convolutions effectively complement grid convolutions, especially in capturing strip-like structures in images [29], such as sperm tails, where they exhibit unique advantages.

This module is introduced at the bottom of the backbone section of the network, immediately following a series of SPDConv and C2f layers. By connecting this module after multiple convolution layers, it refines the features of small sperm targets before further deep feature extraction.

EMA. EMA (efficient multi-scale attention) [30] is an efficient multi-scale attention mechanism that does not require dimension reduction. It can learn effective channel descriptions within convolution operations without reducing the channel dimensions and generates improved pixel-level attention for high-level feature maps.

**Figure 6:**
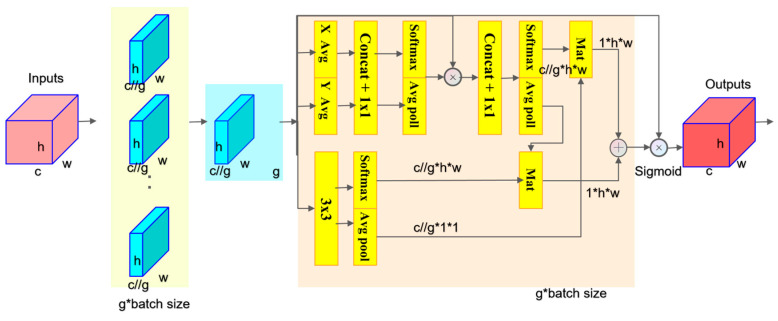
EMA.

The input feature map of dimensions C × H × W (where C is the number of channels, H is the height, and W is the width) is divided into g groups, each with c/g channels. This grouping strategy allows the model to process different parts of the feature map more meticulously, enabling the network to more precisely adjust the contribution of each sub-feature group to the final recognition task and avoiding channel dimension reduction through conventional convolution. The EMA module enhances feature processing by structuring interactions across channels within parallel sub-networks, ensuring spatial semantic features are evenly distributed. It processes each group with horizontal and vertical average pooling to condense width and height dimensions, respectively, capturing features in both directions. A 3 × 3 convolution then enriches the local spatial context. EMA adjusts channel importance while preserving spatial information, followed by re-organizing features: pooled features are concatenated and mixed via a shared 1 × 1 convolution, eliminating the need for reducing dimensions. The 1 × 1 convolution’s output adjusts each channel’s contribution through sigmoid functions. For broader context, it applies global average pooling and transforms global features into a probability distribution with the softmax function, enhancing feature significance. Matrix multiplication merges global and local features, promoting inter-spatial interactions, resulting in attention weights that refine input features. This method, which creates dependencies between channels and spatial locations, has shown effectiveness in various computer vision tasks [31,32]. EMA outputs a weighted feature map matching the input’s dimensions, as shown in Figure 6, effectively leveraging multi-scale and cross-channel information for improved performance.

The goal of the entire EMA module is to enhance the network’s capability to represent input features by capturing spatial details, inter-channel relationships, and cross-spatial position interaction information. This way, the EMA module can improve the model’s ability to capture information across various scales without significantly increasing the computational cost, which is particularly crucial for complex visual tasks, such as in the analysis of high-resolution images or multi-object scenes.

This paper integrates this module into the neck part of the network, following a series of upsampling and Concat (the location of feature fusion) operations. In deep learning networks, the neck section is typically used to process multi-scale features and is positioned between the backbone and detection heads, serving to enhance feature representation before the final object detection.

In summary, the main enhancements of the attention mechanism are as follows:

1. Enhanced feature extraction: Attention mechanisms allow the network to capture and focus on both local and global information, thereby improving the overall feature extraction process. This is crucial for recognizing small and indistinct sperm features in cluttered backgrounds.
2. Improved detection accuracy: By integrating these attention mechanisms, the study has seen significant improvements in key performance indicators such as precision, recall, and mean average precision (mAP). For example, after introducing the SegNext_Attention and EMA modules, precision and recall have noticeably increased, indicating more accurate sperm detection.
3. Better handling of small objects: Attention mechanisms, especially in combination with a small target detection layer (STDL), enhance the network’s sensitivity and accuracy in detecting very small objects. Higher-resolution feature maps retain more detailed spatial information, which is critical for recognizing the complex details of sperm.
4. Balancing local and global information: By strategically placing attention modules between the mid and high layers of the network, the model achieves a balance between focusing on local details and global background information. This reduces the risk of over-generalization and information loss while retaining important features needed for accurate sperm detection.

Detect_DyHead. The dynamic target detection head [33] is a unified detection head that utilizes an attention mechanism, integrating three types of attention across three dimensions to enhance the feature expression of small targets. It not only makes the feature map more sensitive to the scale differences of foreground objects, enhancing the model’s ability to recognize and classify sperm occupying fewer pixels but also focuses more intensively on the spatial location of foreground objects. This prevents the accuracy of target detection and tracking from decreasing due to the occlusion and overlap of targets in scenes with numerous small targets. Furthermore, it can adjust the feature activation state according to the needs of different tasks without increasing additional computational costs.

Scale-aware: An L-dimensional attention mechanism (similar to squeeze-and-excitation networks (SENet)) is applied, where scale-aware attention is utilized for dynamically fusing features across different scales. It is implemented through a 1 × 1 convolutional layer (linear function) and a hard Sigmoid function. The hard Sigmoid function is defined as σ(x) = max (0, min (1, (x + 1)/2)), which serves to constrain the output of scale attention within the [0, 1] range.

Spatial-aware: Detection of targets of different shapes, positions, and angles in spatial locations. Deformable DCN convolution is used for sparse sampling, and an importance factor is set to weight the sampled feature values. Then, features are aggregated across levels at the same spatial position.

Task-aware: Aimed at promoting the joint learning and generalization of different object representations (such as bounding boxes, center points, and corners). The operation of this module can be expressed through the following formula:

(3) $$\pi_{C}(ℱ)\cdot ℱ=\max\left(\alpha^{1}(ℱ)\cdot {ℱ}_{c}+\beta^{1}(ℱ),\alpha^{2}(ℱ)\cdot {ℱ}_{c}+\beta^{2}(ℱ)\right)$$

The feature tensor F is a high-dimensional matrix constructed by the network to process multi-dimensional data, where a feature slice Fc represents the dataset of the cth channel in the multi-channel feature data. The network adjusts the activation level of each channel through a learning function θ(⋅). This function first reduces the dimensionality of the feature data, then connects it through two layers of the network and performs normalization. Finally, the adjusted Sigmoid function controls the data range within [−1, 1], thereby optimizing subsequent learning and recognition tasks.

SPDConv. In the YOLOv8 model, there is extensive use of convolution layers with 3 × 3 sized kernels. However, these modules suffer from two significant flaws: loss of fine-grained information and poorly learned features. In image processing, when using strided convolution, detailed information in the image may be discarded, especially in the detection of low-resolution images or small objects, where crucial details might be lost due to downsizing operations. Due to the loss of fine-grained information, the neural network may not effectively learn features beneficial for the detection of small targets like sperm, leading to decreased model performance as the network fails to capture sufficient information to accurately identify or locate sperm.

This paper employs the SPDConv structure to replace all 3 × 3 convolution layers in the YOLOv model, preventing the loss of fine-grained information of small targets during image processing, especially in the downsampling process. The SPDConv module is employed to augment the model’s capacity to process spatial information, facilitating the distinction of individual sperm within dense clusters. It deepens the feature maps, enabling the model to better interpret complex backgrounds and dense objects.

**Figure 7:**
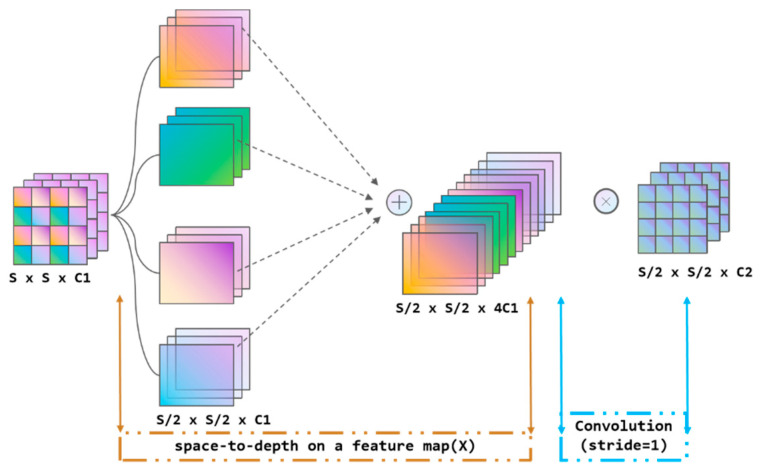
SPDConv.

The SPD-Conv consists of a space-to-depth (SPD) layer and a non-strided convolution layer [34]. The specific structure is illustrated in the following Figure 7. The SPD layer reduces each spatial dimension of the input feature map to the channel dimension while retaining the information within the channel. This is achieved by mapping each pixel of the input feature map to a channel, during which the size of the spatial dimension decreases while the size of the channel dimension increases. The non-strided convolution (Conv) layer, a standard convolution operation performed after the SPD layer, differs from strided convolution in that it does not move across the feature map but performs convolution operations on each pixel or feature mapping. This helps to reduce the potential issue of excessive downsampling in the SPD layer and retains more fine-grained information.

Improving the loss function for small sperm targets. The loss calculation of YOLOv8 includes two branches: the classification and regression branches. The VFL Loss is used as the classification loss, and the DFL Loss + CIOU Loss is used as the regression loss.

CIOU [35] is based on IOU but also considers the distance between the centroids of the two real and predicted boxes (d in the figure), the diagonal distance of the minimum enclosing box (c in the figure, where the minimum enclosing rectangle is the dashed part of the figure), and the consistency of the aspect ratio. DFL [36] proposes to directly regress an arbitrary distribution to model the bounding box, using softmax to implement discrete regression. It derives the integral form of the Dirac distribution to the general form of integral representation for the bounding box, allowing the network to quickly focus on the distribution of positions close to the target location.

CIoU fundamentally remains a metric based on the Intersection over Union (IoU) and is very sensitive to the positional deviation of small objects. Moreover, DFL primarily addresses issues of target occlusion and unclear object boundaries. When there is little to no overlap or complete non-overlap between targets, the modeling method for the target box in this method does not enhance target detection. For sperm detection, however, well-prepared semen samples generally do not present occlusion issues, and sperm are tiny objects. Therefore, this paper utilizes a new distance measurement method to enhance IoU’s sensitivity to small-scale objects, replaces DFL with NWD for bounding box modeling, adjusts the auxiliary box size with a scale factor ratio, and combines MPDIoU loss instead of CIoU to accelerate bounding box regression.

IoU is significantly sensitive to the scale of objects, with minor positional deviations leading to a noticeable drop in IoU for small objects, resulting in inaccurate label assignment. In NWD, a new metric utilizing the Wasserstein distance to measure the similarity of bounding boxes replaces the standard IoU. Specifically, bounding boxes are modeled as two-dimensional Gaussian distributions, and the proposed Normalized Wasserstein Distance (NWD) is used to measure the similarity of the derived Gaussian distributions.

For small objects, their bounding boxes often contain some background pixels since most real objects are not strictly rectangular. In these bounding boxes, foreground pixels and background pixels are concentrated in the center and the edges of the bounding box, respectively. To better describe the weight of different pixels within the bounding box, a rectangular bounding box R = (cx,cy,w,h) can be modeled as a two-dimensional Gaussian distribution N(μ,Σ), where the center pixels of the bounding box have the highest weight, and the importance of pixels decreases from the center towards the edges. The formula is as follows.

(4) $$\mu =\left[\begin{array}{c} c_{x}\\ c_{y} \end{array}\right],\Sigma =\left[\begin{array}{cc} \frac{w^{2}}{4} & 0\\ 0 & \frac{h^{2}}{4} \end{array}\right]$$

The Wasserstein distance from optimal transport theory is used to calculate the distance between distributions. For two two-dimensional Gaussian distributions μ1 = N(m_1_,Σ_1_) and μ2 = N(m_2_,Σ_2_), the second-order Wasserstein distance between μ_1_ and μ_2_ is defined as:

(5) $$W_{2}^{2}\left(\mu_{1},\mu_{2}\right)=∥m_{1}-m_{2}{∥}_{2}^{2}+∥\Sigma_{1}^{1/2}-\Sigma_{2}^{1/2}{∥}_{F}^{2}$$

For Gaussian distributions N_a_ and N_b_ modeled from bounding boxes A = (cx_a_,cy_a_,w_a_,h_a_) and B = (cx_b_,cy_b_,w_b_,h_b_):

(6) $$W_{2}^{2}\left(N_{a},N_{b}\right)=∥\left({\left[cx_{a},cy_{a},\frac{w_{a}}{2},\frac{h_{a}}{2}\right]}^{T},{\left[cx_{b},cy_{b},\frac{w_{b}}{2}\cdot \frac{h_{b}}{2}\right]}^{T}\right){∥}_{2}^{2}$$

However, W_2_ is a distance measure, which cannot be directly used as a similarity measure (i.e., a value between 0 and 1 as IoU). Therefore, we use its exponential form to normalize and obtain a new metric called Normalized Wasserstein Distance (NWD):

(7) $$NWD\left(N_{a},N_{b}\right)=\exp\left(-\frac{\sqrt{W_{2}^{2}\left(N_{a},N_{b}\right)}}{C}\right)$$

The main advantage of the Wasserstein distance is that it can measure the similarity of distributions even when there is no overlap or the overlap is negligible. Moreover, NWD is insensitive to the scale of objects, making it more suitable for measuring the similarity between small objects, where C is a constant closely related to the dataset.

The existing IoU loss function has weak generalization capability in different detection tasks and a slower convergence rate. When the predicted box and the real box have the same aspect ratio, but the width and height values are completely different, the CIoU loss function cannot optimize. In the detection of small sperm targets, the size and morphology of sperm may be relatively uniform, but the actual size in microscopic images may vary due to different sampling depths or imaging conditions. Such changes in scale and aspect ratio might make it difficult for the model to accurately locate and recognize sperm. To address the aforementioned issues and further focus on the detection tasks for small sperm targets, this paper effectively combines the scale factor ratio from Inner-IoU [37], which controls the size of the auxiliary bounding box, with the novel bounding box similarity measure MPDIoU [38] loss function for loss calculation.

The MPDIoU loss function evaluates various factors like area overlap, center point distance, and size deviations to accurately measure bounding box similarity. It aims to minimize the distance between corners of predicted and actual bounding boxes for more effective deep learning training. The Inner-IoU function uses an extra bounding box for IoU loss calculation, adjusting its size with a scaling factor to help the model focus better on smaller targets, which are typically harder to detect. For small sperm detection, this paper suggests resizing the auxiliary bounding box with a scaling factor (1.15) to enhance detection accuracy.

Utilizing MPDIoU to improve the accuracy of bounding box regression, Inner-IoU to accelerate convergence speed, and NWD to address shape differences between bounding boxes enhances the model’s sensitivity to small sperm target detection. In this way, a loss function is provided that not only has the intuitiveness of IoU measurement but also considers bounding box alignment, shape similarity, and the detection of small sperm targets. Below is the algorithm formula combining Inner-MPDIoU with NWD.

B_prd_ and B_gt_ are the coordinates of the predicted and real bounding boxes, w and h are the width and height of the input image, and iou_ratio is set to 0.5.

(8) $${ℬ}_{prd}=\left(x_{1}^{prd},y_{1}^{prd},x_{2}^{prd},y_{2}^{prd}\right),{ℬ}_{gt}=\left(x_{1}^{gt},y_{1}^{gt},x_{2}^{gt},y_{2}^{gt}\right)$$

(9) $$d_{1}^{2}={\left(x_{1}^{prd}-x_{1}^{gt}\right)}^{2}+{\left(y_{1}^{prd}-y_{1}^{gt}\right)}^{2}$$

(10) $$d_{2}^{2}={\left(x_{2}^{prd}-x_{2}^{gt}\right)}^{2}+{\left(y_{2}^{prd}-y_{2}^{gt}\right)}^{2}$$

(11) $$A^{gt}=\left(x_{2}^{gt}-x_{1}^{gt}\right)*\left(y_{2}^{gt}-y_{1}^{gt}\right)$$

(12) $$A^{prd}=\left(x_{2}^{prd}-x_{1}^{prd}\right)*\left(y_{2}^{prd}-y_{1}^{prd}\right)$$

(13) $$x_{1}^{I}=\max\left(x_{1}^{prd},x_{1}^{gt}\right),x_{2}^{I}=\min\left(x_{2}^{prd},x_{2}^{gt}\right),y_{1}^{I}=\max\left(y_{1}^{prd},y_{1}^{gt}\right),y_{2}^{I}=\min\left(y_{2}^{prd},y_{2}^{gt}\right)$$

(14) $$ℐ=\{\begin{array}{ll} \left(x_{2}^{I}-x_{1}^{I}\right)*\left(y_{2}^{I}-y_{1}^{I}\right), & \;\mathrm{if}\;x_{2}^{I}>x_{1}^{I},y_{2}^{I}>y_{1}^{I}\\ 0, & \;\mathrm{otherwise}.\; \end{array}$$

(15) $$IoU=\frac{I}{U},\;\mathrm{where}\;U=A^{gt}+A^{prd}-ℐ$$

(16) $$MPDIoU=IoU-\frac{d_{1}^{2}}{h^{2}+w^{2}}-\frac{d_{2}^{2}}{h^{2}+w^{2}}$$

(17) $${ℒ}_{\mathrm{MPDIoU}\;}=1-MPDIoU$$

(18) $$\text{inter\_area}=\max\left(0,\min\left(x_{2}^{\mathrm{gt}},x_{2}^{\mathrm{prd}\;}\right)-\max\left(x_{1}^{\mathrm{gt}},x_{1}^{\mathrm{prd}\;}\right)\right)*\max\left(0,\min\left(y_{2}^{\mathrm{gt}},y_{2}^{\mathrm{prd}\;}\right)-\max\left(y_{1}^{\mathrm{gt}},y_{1}^{\mathrm{prd}\;}\right)\right)$$

(19) $$w_{\mathrm{gt}}=x_{2}^{gt}-x_{1}^{gt},h_{\mathrm{gt}}=y_{2}^{gt}-y_{1}^{gt},w_{\mathrm{prd}}=x_{2}^{\mathrm{prd}\;}-x_{1}^{\mathrm{prd}\;},h_{\mathrm{prd}}=y_{2}^{\mathrm{prd}\;}-y_{1}^{\mathrm{prd}\;}$$

(20) $${\text{union\_area}=\mathrm{ratio}}^{2}*\left(w_{\mathrm{gt}}{*h}_{\mathrm{gt}}{+w}_{\mathrm{prd}}{*h}_{\mathrm{prd}}\right)\text{-inter\_area}$$

(21) $${\mathrm{IoU}}_{\mathrm{inner}}=\frac{\text{inter\_area}}{\text{union\_area}}$$

(22) $$L_{\text{Inner-MPDIoU}}=L_{MPDIoU}+IoU-IoU^{\mathrm{inner}}$$

(23) $$L=\text{iou\_ratio}\cdot L_{\text{Inner-MPDIoU}}+(1-\text{iou\_ratio})\cdot L_{\mathrm{NWD}}$$

### 3.2. SpermTrack-EVD

In the tracking-by-detection framework for multi-object tracking (MOT), the process involves two main steps: using motion models and state estimation to predict future positions of objects and matching new detections with existing tracks. This involves calculating the Intersection over Union (IoU) and using appearance models for re-identification (Re-ID) to maintain track continuity. This blend of motion and appearance data underpins effective and robust MOT. This paper employs the DeepOCSORT tracking algorithm with an improved visual appearance module for sperm tracking.

DeepOCSORT [39] builds on OC-SORT by dynamically managing the importance of appearance data and incorporating camera motion compensation (CMC) to enhance accuracy in tracking moving objects. It uses a dynamic model for blending appearance information, adjusting its weight based on detection quality. This method boosts the system’s resistance to motion blur and occlusions, ensuring high tracking performance.

To further optimize the tracking algorithm and reduce the ID switch problem, that is, to avoid ID duplication or misidentification in re-identification, this study improves the DeepOCSORT-ReID algorithm. Specifically, we replace the original algorithm’s resnet50 with osnet_ibn_x1_0 as the backbone network, aiming to optimize the tracking algorithm by learning full-scale feature representations. The model is trained using a sperm dataset, making the tracking algorithm more targeted toward small sperm objects. This upgrade is crucial for maintaining the identity of sperm cells during tracking, especially when they move close to each other or overlap.

OS-Net is a lightweight CNN with a design focused on multi-scale feature capture through various convolutional streams of different sizes. These streams increase linearly in size, enabling the network to handle features at multiple scales. A key component, the unified aggregation gate (AG), dynamically blends these features by generating channel weights based on the input, facilitating dynamic fusion of scales. This architecture adapts to different image needs by prioritizing certain feature streams, allowing for comprehensive feature representation across all scales.

**Figure 8:**
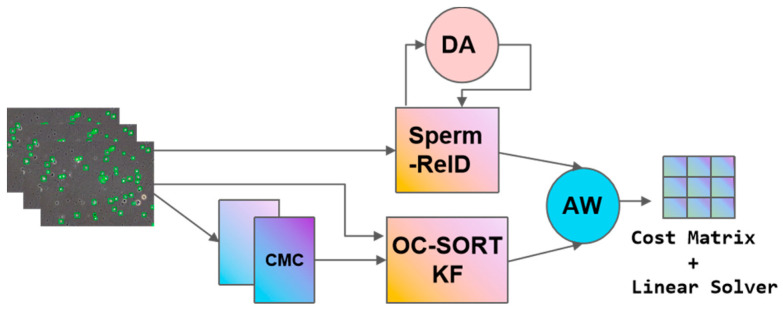
SpermTrack-EVD.

This replacement aims to extract richer Re-ID features to reduce misidentification and tracking errors, optimize the algorithm to reduce ID switches, and enhance the continuity of tracking. The improved DeepOCSORT tracking algorithm is illustrated in Figure 8.

## 4. Evaluation Metrics

In evaluating the improved algorithm, two main aspects were considered: sperm detection and sperm tracking. The following evaluation metrics were used to assess the algorithm’s performance in various aspects:

Detection. This paper evaluates the detection algorithm using Precision (P), Recall (R), and Mean Average Precision (mAP) metrics, forming a framework to assess algorithm accuracy and coverage in identifying correct detections. Precision measures the proportion of correct positive detections out of all positive detections made by the algorithm. Recall is the proportion of correct positive detections out of all actual positives. mAP0.5 and mAP0.5:0.95 are based on varying IoU thresholds, which determine how well predicted bounding boxes match actual ones. mAP0.5 accounts for detections with an IoU of at least 0.5, meaning predictions with an IoU below 0.5 are not considered correct. mAP0.5:0.95 averages AP for IoU thresholds from 0.5 to 0.95, increasing in steps of 0.05, making it a stricter measure.

(24) $$P=\frac{TP}{TP+FP}$$

(25) $$R=\frac{TP}{TP+FN}$$

(26) $$\mathrm{mAP}0.5=\frac{1}{N}\overset{n}{\underset{i=1}{\sum }}{\mathrm{AP}}_{0.5,i}$$

(27) $$\mathrm{mAP}0.5:0.95=\frac{1}{N}\overset{n}{\underset{i=1}{\sum }}\frac{1}{10}\overset{0.95}{\underset{t=0.5}{\sum }}{\mathrm{AP}}_{t,i}$$

TP (True Positives) represents the number of samples correctly predicted as positives, while FP (False Positives) refers to the number of negative samples incorrectly marked as positives. FN (False Negatives) denotes the number of positive samples wrongly predicted as negatives. N is the total number of categories, and AP_t,i_ is the average precision for the ith category at an IoU threshold t.

Tracking. MOTA is a multi-object tracking accuracy metric that comprehensively considers detection accuracy, missed detections, false detections, and identity switches. MOTP measures the average positional accuracy of correctly tracked targets. IDF1 is a metric that evaluates a tracker’s ability to maintain target identity consistency based on the harmonic mean of identity precision and recall. HOTA is a newer metric that balances the performance of detection and identity matching, with the formula as follows.

(28) $$\mathrm{HOTA}=\sqrt{\frac{\sum_{\alpha \in A}\mathrm{Det}A(\alpha )\cdot A\mathrm{ssA}(\alpha )}{\left|TP+FN+FP\right|}}$$

where DetA(α) denotes detection accuracy at a given threshold α, and AssA(α) denotes association accuracy at a given threshold α.

## 5. Experiments

### 5.1. Dataset

The improved detector was trained on the VISEM-Tracking [40] dataset. The VISEM-Tracking dataset is a dataset focused on human sperm tracking, co-created by researchers from SimulaMet and OsloMet in Norway. It was collected using specialized equipment for capturing human sperm videos, including an Olympus CX31 microscope equipped with 400× magnification capabilities and a UEye UI-2210C camera produced by IDS Imaging Development Systems. The manufacturer of the Olympus CX31 microscope is Olympus Corporation, headquartered in Tokyo, Japan. The UEye UI-2210C camera is manufactured by IDS Imaging Development Systems GmbH, based in Obersulm, Germany. These devices were mounted on the microscope for video recording purposes. To ensure the quality of the samples during observation and recording, the samples were placed on a heated microscope stage with temperature control set to 37 degrees Celsius, aiming to mimic the natural environment within the human body and to ensure that the activity state of the sperm closely approximates its natural physiological condition. This high-standard equipment configuration and precise environmental control allow the VISEM-Tracking dataset to provide high-quality, manually annotated sperm motion videos, including bounding box coordinates for each spermatozoon and sperm characteristics analyzed by domain experts. Thus, it offers a valuable data resource for computer-assisted sperm analysis (CASA) and the assessment of sperm motion and kinetics based on machine learning approaches.

The sperm videos in this dataset are recorded using a WHO-recommended technique that prepares a wet slide with a fixed depth of 20 μm, allowing sperm to move freely while minimizing out-of-focus movements during brief recordings. The setup, including the use of high-speed cameras like the UEye UI-2210C, confines the analysis to primarily two-dimensional motion within this shallow depth. This approach significantly reduces the impact of movements along the *Z*-axis, ensuring that the analysis predominantly focuses on speed and linearity in the focal plane despite not capturing complex three-dimensional behaviors like spinning or tilting.

The dataset contains 20 video recordings, each lasting 30 s, for a total of 29,196 frames, showcasing wet-prepared sperm samples. Each sperm in the dataset is labeled with different tags, including ‘pathsperm’, ‘cluster’, and ‘small or pinhead’. Additionally, full files of each video (.mp4 format) are provided, with all bounding box coordinates given in YOLO format.

**Figure 9:**
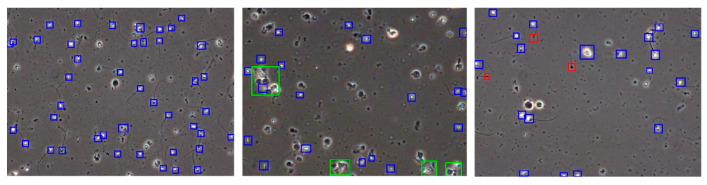
Video frames of wet semen preparations with corresponding bounding boxes. Top: large images showing different classes of bounding boxes, blue—sperm, green—sperm cluster, and red—small or pinhead sperm.

The pinhead category consists of spermatozoa with abnormally small, black heads within the view of the microscope. The cluster category consists of several spermatozoa grouped together. Sample annotations are presented in Figure 9. The blue boxes represent normal spermatozoa cells which constitute the majority of this dataset and are also biologically most relevant. The green boxes represent sperm clusters where few spermatozoa cells are clustered together, making it hard to annotate sperm cells separately. The red color boxes represent small or pinhead spermatozoa which are smaller than normal spermatozoa and have very small heads compared to a normal sperm head.

The VISEM-Tracking dataset is relatively large in terms of data volume. During training, the original dataset was divided into training, testing, and validation sets, with 19,530 images for the training set, 5580 images for the test set, and 2790 images for the validation set.

To improve the accuracy of extracting sperm appearance features in tracking, the visual appearance module in the tracking algorithm was jointly trained on the VISEM-Tracking dataset and the MHSMA dataset [41], with part of the data from the MHSMA dataset used to augment the VISEM-Tracking dataset. The MHSMA dataset consists of 1540 unstained grayscale sperm images, each containing one sperm, with image resolutions of either 64 × 64 pixels or 128 × 128 pixels. These images were collected using low-magnification microscopes (600× and 400×). The MHSMA dataset is characterized by each sperm’s different parts (head, acrosome, vacuole, tail, and neck) being annotated as normal (positive) or abnormal (negative) by experts in the field of sperm morphology analysis. Since we only need to classify sperms as “normal sperm”, “pinhead sperm”, and “clustered sperm”, we categorized sperms with abnormalities in the head, acrosome, or vacuole as “pinhead sperms” and added them to the corresponding classes in the VISEM-Tracking dataset along with MHSMA dataset sperms that are normal in all parts: head, acrosome, vacuole, tail, and neck.

Finally, the augmented VISEM-Tracking dataset was converted into the format required for training the visual appearance model. The details are shown in Table 1. The training set is a collection of data utilized for training the model, enabling it to learn the identification of different object categories. The query set is used to assess the model’s performance on unknown data, essentially serving to validate the model’s ability to recognize or retrieve information from unseen data during training. The gallery set is used during the execution of queries, from which the model attempts to retrieve matching items. The model strives to find the images within the gallery set that best match the images from the query set.

The proposed enhanced YOLOv8 small target detection algorithm and the improved DeepOCSORT tracking algorithm were evaluated on the VISEM-Tracking dataset.

### 5.2. Experimental Settings

The experimentation was carried out on a high-performance computing setup encompassing an Intel(R) Xeon(R) Silver 4214R CPU operating at 2.40 GHz and equipped with twelve vCPUs. An NVIDIA GeForce RTX 3080 GPU endowed with 12 GB of VRAM, was employed. The system boasted a 90 GB RAM configuration. These experiments were conducted on an Ubuntu 20.04 operating system, utilizing PyTorch 1.11.0 as the chosen deep learning framework, and GPU acceleration was facilitated through CUDA version 11.3. Python 3.8 served as the programming language.

The training process utilized the Stochastic Gradient Descent (SGD) optimizer [42], with an initial learning rate set to 0.01, which remained unchanged throughout the training period. Additionally, to accelerate convergence speed and enhance stability during training, we set the momentum to 0.937. Momentum assists the optimizer in accumulating speed in the same direction as the gradient, reducing oscillations, thereby speeding up the learning process and helping to avoid falling into local minima. To improve optimization efficiency on different parameters and prevent overfitting, we categorized model parameters into three groups for special configuration. The first group contained 79 weight parameters, with no weight decay applied (decay = 0.0), aiming to allow the model more freedom to learn on these layers; the second group contained 109 weight parameters, with a weight decay of 0.0005 applied to fine-tune the model and mitigate the risk of overfitting; the third group contained 110 bias parameters, also without weight decay, as bias parameters generally have a lesser impact on model overfitting. Moreover, to accommodate input images of different resolutions, image sizes in the training and validation sets were set to 640 pixels. Lastly, to speed up data loading and enhance training efficiency, we employed four data-loading worker threads, which help prepare and supply training data more quickly. A batch size of 16 was adopted for training, extending across 100 epochs. The practice of disabling Mosaic in the last 10 epochs was adopted, with the total number of epochs for training increased from 300 to 500. We detail all hyperparameters and model parameters in the appendix of the article as Appendix A and Appendix B. 

### 5.3. Experimental Results

**Figure 10:**
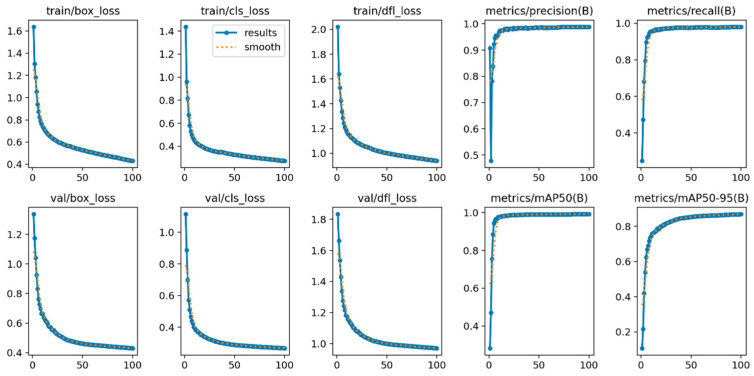
Training results of Sperm YOLOv8-E.

Sperm Small Target Detection Results. The training results of SpermYOLOv8-E are shown in Figure 10, displaying the changes in training loss, validation loss, precision, recall, mAP@0.5, and mAP@0.5:0.95 over time. The training and validation losses decreased over time, indicating an improvement in the model’s learning effect. Precision, recall, mAP@0.5, and mAP@0.5:0.95 showed an upward trend, indicating an improvement in the model’s detection performance. These results demonstrate the effectiveness of the sperm detection model in accurately identifying sperm under a microscope.

As shown in Table 2, SpermYOLOv8-E was evaluated against the benchmark YOLOv8 network. In tracking-by-detection (TBD), the precision of the detection model is crucial. This study achieved significant improvements in key performance indicators such as accuracy, recall, and mAP through systematic enhancements to the YOLOv8 model. Enhancements include the integration of SPDConv, Detect_DyHead, the introduction of STDL, and the integration of Inner-MPDIoU/NWD.

The introduction of the SPDConv module improved precision from 0.85 to 0.884, recall from 0.821 to 0.857, mAP0.5 from 0.828 to 0.875, and mAP0.5:0.95 from 0.634 to 0.744. This indicates that SPDConv, by increasing the depth of feature maps, can more effectively process spatial information, enhancing the model’s understanding of complex backgrounds. The addition of SegNext_Attention and EMA modules also improved model accuracy to some extent. The combination of these two attention mechanisms provides multi-scale and channel feature fusion, significantly improving the model’s ability to understand different feature layers. With the addition of the small target detection layer, model precision further improved to 0.956, recall to 0.947, mAP0.5 to 0.927, and mAP0.5:0.95 to 0.805. STDL, specifically optimized for small target detection, introduces more delicate features at a lower level of feature extraction, significantly proving the importance of STDL in enhancing the model’s ability to detect small targets. The application of the Detect_DyHead module raised precision to 0.971, recall to 0.956, mAP0.5 to 0.931, and mAP0.5:0.95 to 0.827. As a dynamic detection head module, Detect_DyHead, by improving the feature pyramid, provided more precise detection capabilities for different scale feature layers, especially in target detection with large scale variations. The integration of Inner-MPDIoU/NWD achieved the highest values in all performance metrics, with precision reaching 0.982, recall reaching 0.962, and mAP0.5 reaching 0.951. Inner-MPDIoU improved the IoU calculation method by considering the overlap between the predicted and actual boxes. NWD, as a novel loss function, optimized the model’s localization accuracy by considering the distribution difference between the predicted and actual boxes. The combination of these two techniques greatly improved the model’s detection precision for small targets, with all these results being the highest values in the table, fully demonstrating the effectiveness of Inner-MPDIoU/NWD in enhancing detection performance.

To ensure the comprehensiveness and interpretability of our experimental results, we have selected the three most effective modules for ablation experiments. We chose these three modules because they have demonstrated significant improvements in enhancing sperm detection and tracking functionality.

To comprehensively evaluate the contributions of these modules, we designed the following combinations for ablation experiments: spermE-STDL: To evaluate the contribution of STDL in detecting small targets, Includes only STDL (Small Target Detection Layer) + Detect_DyHead + Inner-MPDIoU/NWD; spermE-Attention: To evaluate the role of the attention mechanism in improving model performance, Includes only SegNext_Attention (MSCA Module) + EMA (Efficient Multi-Scale Attention) + Detect_DyHead + Inner-MPDIoU/NWD; spermE-SPDConv: To evaluate the contribution of SPDConv in retaining fine-grained information and detecting small targets, Includes STDL + SegNext_Attention (MSCA Module) + EMA (Efficient Multi-Scale Attention) + Detect_DyHead + Inner-MPDIoU/NWD; spermE-STDL-Attention: To evaluate the combined effect of STDL and the attention mechanism,spermE-STDL-SPDConv: To evaluate the combined effect of STDL and SPDConv,spermE-Attention-SPDConv: To evaluate the combined effect of the attention mechanism and SPDConv.

We believe that through these combined ablation experiments, we can more clearly demonstrate the performance improvements of each module and their combinations in sperm detection and tracking, thereby more comprehensively validating the effectiveness of our proposed method.

Based on the image of the ablation study table (Table 3), the effectiveness of the three modules (STDL, SPDConv, and Attention Mechanism) and their combinations can be analyzed as follows:

Individual Module Effectiveness: spermE-STDL: Shows high precision and recall, indicating that the Small Target Detection Layer (STDL) effectively improves the accuracy of detecting small targets like sperm. spermE-Attention: This model has slightly lower precision and recall compared to spermE-STDL, but the attention mechanisms still contribute positively, suggesting usefulness in capturing global contextual information that enhances feature expression. spermE-SPDConv: The performance metrics for SPDConv are decent, which supports its role in preserving fine-grained information and improving spatial resolution.

Combined Modules Effectiveness: spermE-STDL-Attention: Combines STDL with attention mechanisms, showing an increase in both precision and recall compared to the models with individual components, indicating that the combination of detailed detection layers and global context capturing is beneficial. spermE-STDL-SPDConv: This combination also performs well. This indicates that the combination of enhancing both the spatial resolution (through SPDConv) and the semantic depth of the feature maps (through STDL) is highly effective for consistent performance across varying degrees of detection precision. spermE-Attention-SPDConv: While this model shows a small drop in precision compared to other combined models, it maintains high recall and mAP0.5, suggesting that the combination is effective, particularly in scenarios requiring detailed contextual information and fine-grained feature preservation.

General Observations: The spermE-STDL-SPDConv model demonstrated the highest performance, with the STDL’s capability to capture fine details of small targets being supported by SPDConv, which further enhances these features through improved spatial resolution. This dual enhancement allows the model to maintain high detection accuracy across different IoU thresholds, whether it be the more lenient 0.5 IoU threshold or the stricter 0.5:0.95 IoU range.

**Figure 11:**
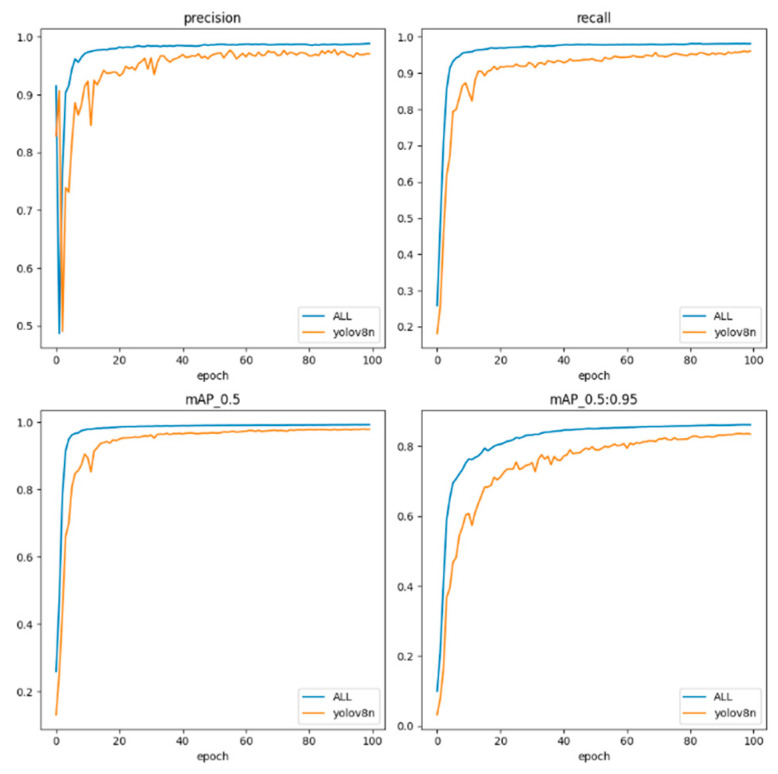
Training results of SpermYOLOv8-E and the benchmark YOLOv8.

Figure 11 vividly presents the significant effects of the targeted improvements made for sperm small target detection through a direct comparison between the final model and the benchmark YOLOv8 network; ALL in the figure represents SpermYOLOv8-E.

**Figure 12:**
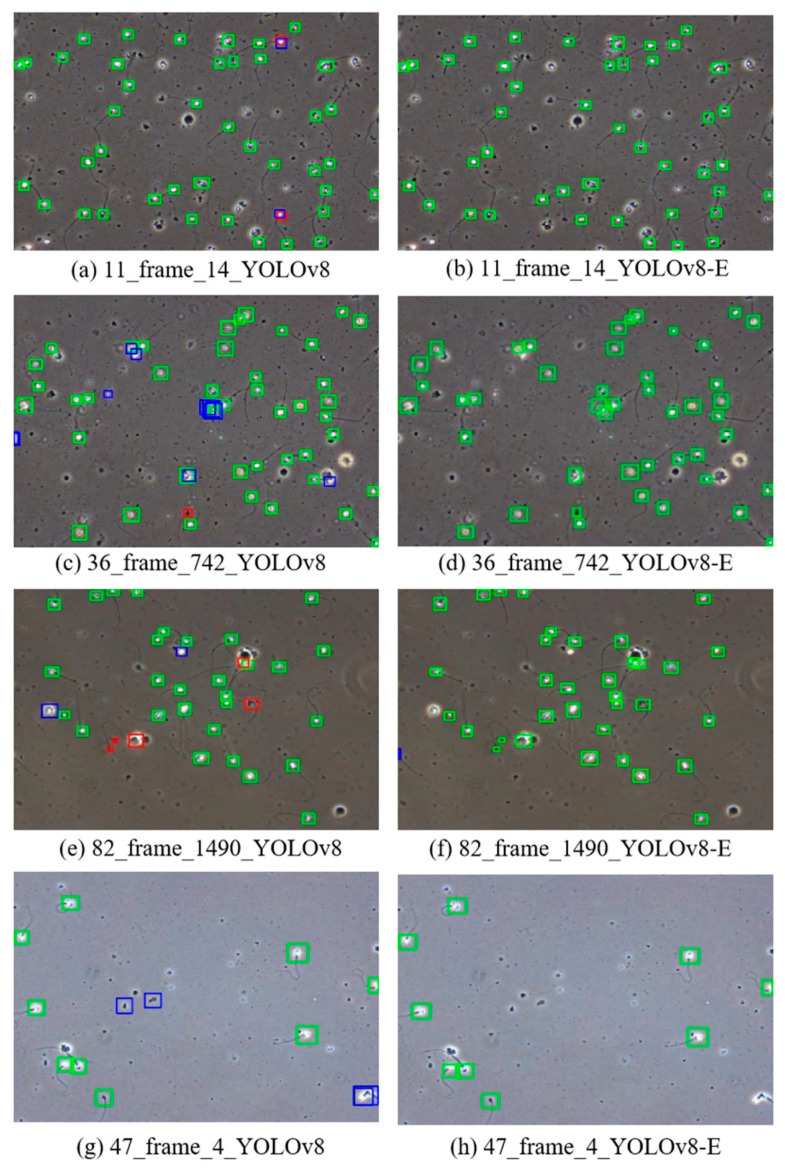
The detection results of the benchmark YOLOv8 (**left**) and SpermYOLOv8-E (**right**).

Figure 12 displays the detection results of YOLOv8 and the improved model on semen sample images. These images comprehensively showcase the comparison of detection results in dense sperm scenes and scenes with impurities. Green boxes indicate correct predictions of both location and category, red boxes indicate missed detections, and blue boxes indicate false detections.

The improved YOLOv8 model demonstrates higher detection accuracy, with an increase in the number of green boxes and a decrease in the instances of red and blue boxes compared to the benchmark model. Especially in dense sperm scenes, the improved model can more effectively identify individual sperm, reducing the number of missed and false detections. For samples containing impurities, the improved YOLOv8 model shows a better ability to distinguish between sperm and impurities, as evidenced by the reduction of blue boxes.

**Figure 13:**
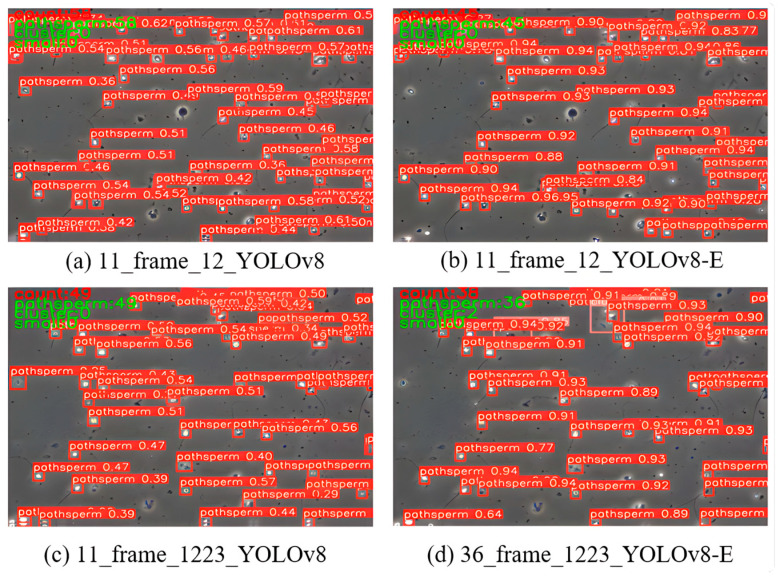
The detailed detection results of the benchmark YOLOv8 (**left**) and SpermYOLOv8-E (**right**).

Figure 13 presents detailed detection results of YOLOv8 and the improved model on semen sample images. In these images, bounding boxes annotate the detected sperm, with each bounding box accompanied by a category label and a confidence score to indicate the model’s certainty in its detection.

The benchmark YOLOv8 model exhibited a high detection sensitivity in Figure 8, but this sensitivity was not paired with high selectivity, leading the model to misidentify non-sperm elements as sperm, which could result in significant errors in sperm counting and analysis. Furthermore, the model’s failure to recognize clusters of sperm further illustrates its limitations when dealing with complex samples, possibly due to the algorithm’s inability to learn features sufficient to distinguish sperm from impurities or limitations in detecting closely connected objects. These issues highlight that, despite the benchmark model’s confidence in each detected target, this confidence is not robust and does not accurately reflect reality, thereby emphasizing the need for precision and specificity in optimizing sperm detection models. In Figure 13, the improved model generally scores higher in confidence, indicating significant improvements in detection accuracy and specificity.

Sperm detection models are vitally important for the fields of clinical medicine and reproductive science research. Sperm quality and quantity are key indicators of male fertility, directly affecting the success rates of assisted reproductive technologies such as in vitro fertilization (IVF) and artificial insemination (AI). In dense sperm scenes, conventional detection methods struggle to accurately differentiate and count, whereas an efficient sperm detection model can overcome this challenge, identifying and distinguishing individual sperm in complex backgrounds, providing doctors and researchers with more precise assessments of sperm count and activity. Additionally, common non-sperm elements in semen samples, such as cellular debris and crystals, can affect the accuracy of sperm counts. Advanced sperm detection models can effectively differentiate between actual sperm and these impurities, avoiding miscounts and thus enhancing overall detection accuracy, which is significant for diagnosing infertility and increasing the success rates of assisted reproductive technologies. Therefore, the model’s good performance in these scenarios means it is more reliable in real-world applications, offering users faster and more accurate analysis results, thereby improving diagnostic efficiency and research quality.

Sperm Tracking Results. To evaluate the effectiveness of the algorithm, we compared the common YOLOv8n detector with our SpermYOLOv8-E detector using the same tracking algorithm; under the condition of jointly using SpermYOLOv8-E as the detector, SpermTrack-EVD tracker, DeepOCSORT, OC-SORT, and BoT-SORT were compared.

Table 4 lists the pedestrian tracking results of the SpermTrack-EVD tracker on the VISEM-Tracking dataset. The analysis shows that the performance of the YOLOv8-based detector is lower than that of SpermYOLOv8-E across all metrics. Specifically, the MOTA of YOLOv8 is 59.782%, while SpermYOLOv8-E significantly improved to 71.167%, demonstrating significant improvements in reducing mismatches and missed detections. Similarly, IDF1 increased from 61.143% to 69.784%, and IDs reduced from 828 to 542, indicating that the improved model performs better in maintaining target identity consistency. In the HOTA metric, SpermYOLOv8-E surpassed YOLOv8 with a score of 74.303%, further proving the comprehensive performance improvement in detecting and tracking targets. The AssA and AssR metrics related to target association also showed a similar trend, with SpermYOLOv8-E achieving an association accuracy of 68.552%, compared to the benchmark model’s 59.902%, and improving the association recall rate from 62.735% to 70.424%.

In summary, the data in the table indicate that, in complex sperm tracking scenarios, SpermYOLOv8-E outperforms the benchmark YOLOv8 model in all key performance indicators. These improvements are likely due to customized model adjustments for specific tasks (such as sperm tracking), including more effective feature extraction, more precise target localization, and stronger identity preservation mechanisms. These results highlight the importance of computer vision models specially developed for semen analysis and tracking and their potential to enhance the accuracy of automated semen analysis systems.

Table 5 shows that, compared to other methods, our approach excels in multiple metrics, including MOTA, IDF1, MOTP, HOTA, and IDs scores. These results validate the effectiveness of our method in achieving precise and robust multi-object tracking.

To achieve accurate multi-target sperm detection and tracking while maintaining real-time processing capabilities for subsequent clinical applications, the proposed algorithm exhibits the following performance metrics when processing video frames: an average preprocessing time of 1.2 ms per frame, an average inference time of 19.9 ms per frame, an average postprocessing time of 1.1 ms per frame, and an average tracking time of 72.5 ms per frame. Consequently, the total average processing time per frame is 94.7 ms. This equates to a processing rate of approximately 10.55 frames per second (1000 ms/94.7 ms ≈ 10.55 fps). These results indicate that the algorithm is highly efficient in handling video data, thus enabling near real-time sperm detection and tracking, which is crucial for clinical applications.

**Figure 14:**
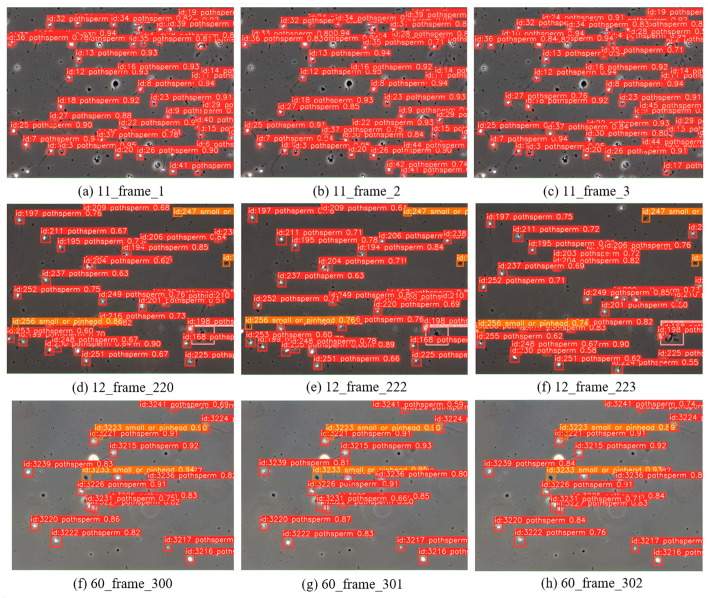
The sperm tracking effect of three consecutive frames in the video.

Visualization of Tracking Results. Figure 14 presents the tracking results of the proposed algorithm on the VISEM-Tracking dataset. The results demonstrate the effectiveness of the proposed method in complex traffic scenarios.

**Figure 15:**
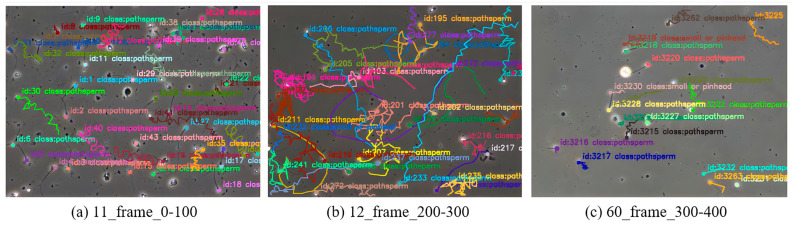
The sperm tracking movement trajectory diagram.

In the video, the movement trajectory of each sperm is drawn, with different colors representing different sperms. The trajectories are displayed from the beginning to the end, ensuring a clear visualization of the sperm movement paths and their ID consistency. Figure 15 shows the sperm video tracking trajectory. (11_frame_0–100 indicates the 11th sperm video, showing the tracking trajectory from frame 0 to frame 100).

## 6. Conclusions

This study successfully combined the enhanced YOLOv8 small target detection algorithm (SpermYOLOv8-E) with the improved DeepOCSORT tracking algorithm (SpermTrack-EVD), proposing a comprehensive sperm tracking algorithm. The algorithm was trained and tested on the VISEM-Tracking dataset, proving its effectiveness in sperm detection and tracking.

The model proposed in this study can directly analyze prepared wet sperm microscopic videos without the need for sperm staining, automatically extracting temporal sequence data of all sperm in the video. This temporal sequence data records in detail the motion trajectory and morphological classification of each sperm in the video. By accurately and stably tracking the motion trajectory of sperm, the model can provide information on the position and speed of sperm at different time points in the video sequence. This functionality not only facilitates the efficient assessment of sperm motility characteristics and sperm concentration but also provides significant support for the standardized assessment of male fertility. Moreover, the model’s ability to automatically evaluate sperm morphology enables it to classify sperm based on morphological features as “normal sperm”, “pinhead”, and “cluster”. The application of this technology in clinical settings is expected to significantly reduce the workload of embryologists in the sperm morphology analysis process while improving the accuracy and efficiency of abnormal sperm detection.

Despite the significant achievements of this study, there are still some challenges and limitations in the development of sperm-tracking algorithms that need further exploration and resolution in future research:

1. Model lightweighting and optimization: Although the current algorithm has improved in accuracy, the complexity of the model and the consumption of computational resources remain high. Future work could explore more lightweight model architectures to reduce the running cost and improve the real-time performance of the algorithm while maintaining high accuracy.
2. Robustness of the tracking algorithm: Successful tracking of sperm in complex backgrounds or high sperm density conditions still faces challenges. Future research could further improve the tracking algorithm to enhance its adaptability and robustness in complex environments.
3. We recognize the importance of further validating our models on other large datasets. While our current research primarily involves the VISEM-Tracking dataset, future work will include multi-dataset validation, testing our algorithms on other large datasets, including the Face Tracking dataset [45], to ensure stability and accuracy under different conditions. We also plan to extend the application scenarios of our algorithms, not limited to sperm detection and tracking but also applicable to other small target detection tasks, such as cell tracking and microscopic image analysis.
4. In our study, we utilized the VISEM-Tracking dataset to classify sperm based solely on morphological characteristics. This classification does not include motility characteristics, and the categories based on morphology are relatively limited. We acknowledge the limitations of our current classification method and propose several future improvements.

Integration of Motility Characteristics

Future algorithms can integrate sperm motility characteristics, such as progressive motility and vitality, to enhance classification accuracy. Given the importance of motility in assessing sperm quality, we plan to develop and incorporate motion analysis algorithms to identify sperm vitality and movement patterns. By including motility data, we can achieve a more comprehensive evaluation of sperm function and health.

Expansion of Classification Categories

Our goal is to include more sperm morphological categories, such as double-headed sperm, bent-tail sperm, and short-tail sperm, to refine the classification standards. This approach will allow for a more detailed analysis of morphological abnormalities, thereby improving diagnostic precision. Expanding the categories will also help in identifying specific morphological defects that may correlate with infertility issues.

Combining Multimodal Data

By combining morphological and motility characteristics, we can provide a holistic assessment of sperm quality and health. Integrating multiple data sources will enhance the robustness and accuracy of sperm classification algorithms. For instance, combining video data with movement trajectory analysis and microscopic image morphology analysis can offer a more comprehensive evaluation of sperm quality. This multimodal approach will leverage the strengths of various data types to provide a richer understanding of sperm function.

5. Although powerful, attention mechanisms also have limitations in cases where sperm features are not prominent for reasons such as low contrast and resolution, cluttered background, and overlap and clustering. Since the VISEM-Tracking dataset has been standardized and prepared, these problems are not prominent. To address these issues in other datasets and clinical trials in the future, we propose the following solutions and improvement goals.

Data augmentation and synthetic data

Enhancing the training dataset with augmented or synthetic data generated by techniques such as Generative Adversarial Networks (GANs) can improve the model’s generalization ability and its ability to detect less obvious features.

Temporal analysis of video data

For sperm tracking in video data, incorporating temporal information may be helpful. Using Recurrent Neural Networks (RNNs) or Long Short-Term Memory networks (LSTMs) can help understand movement patterns and improve detection accuracy over time.

6. Considering the diversity of future datasets, we will consider targeting algorithms for weakly supervised object detection. This can be achieved by training the model using unlabeled or partially labeled data, using weak labels to infer the rough position of objects, thereby reducing reliance on precisely labeled data. For example, using image-level labels to predict the presence of targets, and then combining local clues to infer the approximate position of targets. In tracking algorithms, more emphasis is placed on the structured relationships between targets, using algorithms such as Minimum Spanning Tree (MST) to construct and maintain correlations between targets. In the case of significant changes in sperm dynamics, maintaining spatial and temporal consistency between targets can improve tracking stability and accuracy.

By addressing the above challenges, future research is expected to further optimize and refine sperm tracking algorithms, providing more precise and efficient technical support for the diagnosis and treatment of male infertility.

## Figures and Tables

**Table 1:** Data format for visual appearance model training.

Subset	Ids	Images
Train	3	459,291
Query	3	65,110
Gallery	3	128,707

**Table 2:** Ablation Study Evaluation of Improved Module Efficacy.

Method	Precision	Recall	mAP0.5	mAP0.5:0.95	Parameters	Size/MB	FLOPs/G
YOLOv8n	0.85	0.821	0.828	0.634	3,011,433	6.2	8.2
+SPDConv	0.884	0.857	0.875	0.744	4,739,433	9.7	11.7
+SPDConv +SegNext_Attention	0.901	0.879	0.895	0.766	4,734,391	9.9	11.7
+SPDConv +SegNext_Attention + STDL	0.956	0.947	0.927	0.805	4,996,740	10	16.1
+SPDConv +SegNext_Attention + STDL +Detect_DyHead	0.971	0.956	0.931	0.827	5,325,443	10.7	21.7
+SPDConv +SegNext_Attention + STDL +Detect_DyHead + EMA	0.966	0.951	0.944	0.838	5,326,931	11.2	24.2
+SPDConv +SegNext_Attention + STDL +Detect_DyHead + EMA +Inner-MPDIoU/NWD	0.982	0.962	0.951	0.832	5,326,931	11.2	24.2

**Table 3:** Ablation study assessment of efficacy of three important modules.

Model	Precision	Recall	mAP0.5	mAP0.5:0.95	Parameters	Size/MB	FLOPs/G
spermE-STDL	0.932	0.909	0.874	0.679	3,512,488	7	19.8
spermE-Attention	0.863	0.858	0.872	0.663	3,591,045	7.6	10.4
spermE-SPDConv	0.915	0.877	0.886	0.760	5,219,237	10.4	13.3
spermE-STDL-Attention	0.932	0.911	0.903	0.721	3,607,928	10.6	20.4
spermE-STDL-SPDConv	0.971	0.945	0.918	0.799	5,268,136	10.9	23.7
spermE-Attention-SPDConv	0.907	0.894	0.919	0.773	5,319,045	11	13.9

**Table 4:** Tracking Performance Comparison of SpermYOLOv8-E and Benchmark YOLOv8 on VISEM-Tracking Using SpermTrack-EVD.

Detector	MOTA	MOTP	IDF1	HOTA	AssA	AssR	IDs
yolov8n	59.782	81.387	61.143	62.322	59.902	62.735	828
SpermYOLOv8-E	71.167	90.879	69.784	74.303	68.552	70.424	542

**Table 5:** Performance comparison with preceding SOTAs on VISEM-Tracking.

Detector	MOTA	MOTP	IDF1	HOTA	IDs
SpermTrack-EVD	71.167	90.879	69.784	74.303	542
DeepOCSORT	64.053	89.387	62.805	66.873	896
OC-SORT [43]	63.771	88.992	60.362	65.431	1394
BoT-SORT [44]	61.272	85.465	58.193	64.353	1728

## Data Availability

We express our heartfelt gratitude to the research team from SimulaMet Oslo, Norway, and OsloMet Oslo, Norway, especially the lead author, Vajira Thambawita. Their diligent efforts allowed us access to the VISEM-Tracking dataset, a unique dataset for tracking human spermatozoa. Dataset link: https://zenodo.org/record/7293726.

## Appendix A. List of Hyperparameters

1. Learning rate and momentum parameters

parameter	value	explain
lr0	0.01	Initial learning rate (initial value of 0.01).
lrf	0.01	Final learning rate (initial learning rate multiplied by this value). The final learning rate is 0.01 * 0.01 = 0.0001.
momentum	0.937	Momentum of the SGD optimizer. Momentum is used to accelerate convergence and reduce oscillations.
weight_decay	0.0005	The weights of the optimizer are attenuated to prevent overfitting with a value of 5 × 10^−4^.

2. Preheating parameters

parameter	value	explain
warmup_epochs	3.0	Number of cycles for warm-up training. During the warm-up period, the learning rate is gradually increased from a smaller value to a set initial learning rate.
warmup_momentum	0.8	Initial momentum value during warm-up.
warmup_bias_lr	0.1	Initial bias learning rate during warm-up.

3. Loss function gain

parameter	value	explain
box	7.5	Border regression loss gain coefficient. Controls the weight of the border regression loss.
cls	0.5	Category loss gain factor. Scales the weight of the category loss according to the number of pixels.
dfl	1.5	Distributed focus loss gain coefficient. Weights used for distributed focal length regression.

4. Batch size

parameter	value	explain
nbs	64	Nominal batch size. The batch size used to normalize the learning rate and other hyperparameters.

5. Image enhancement parameters

parameter	value	explain
hsv_h	0.015	Image HSV hue enhancement. Percentage of the range of hue changes.
hsv_s	0.7	Image HSV saturation enhancement. Percentage of saturation change range.
hsv_v	0.4	Image HSV value (brightness) enhancement. Percentage of the range of brightness changes.
translate	0.1	Image panning enhancement. Percentage of panning range (+/−).
scale	0.5	Image zoom enhancement. Percentage of zoom range (+/−).
fliplr	0.5	Image left/right flip enhancement. The probability of flipping left and right.
mosaic	1.0	Image mosaic enhancement. Probability of applying mosaic enhancement.

Enhancement methods	parameter	explain
Blur	*p* = 0.01, blur_limit = (3, 7)	Fuzzy enhancement, with a 1% probability of application, uses fuzzy kernels in the range of 3 to 7.
MedianBlur	*p* = 0.01, blur_limit = (3, 7)	Median fuzzy enhancement, with a 1% probability of application, uses a median fuzzy kernel in the range of 3 to 7.
ToGray	*p* = 0.01	Grayscale enhancement with a 1% probability of converting the image to grayscale.
CLAHE	*p* = 0.01,clip_limit = (1,4.0), tile_grid_size = (8, 8)	Adaptive histogram equalization enhancement with 1% probability of application, contrast limits from 1 to 4, and plot size of 8x8.

## Appendix B. List of Model Layer Parameters

nc: 3 # number of classes scale ‘n’ # [depth, width, max_channels] n: [0.33, 0.25, 1024]

backbone: # [from, repeats, module, args] - [−1, 1, Conv, [64, 3, 2]] # 0-P1/2 - [−1, 1, SPDConv, [128]] # 1-P2/4 - [−1, 3, C2f, [128, True]] - [−1, 1, SPDConv, [256]] # 3-P3/8 - [−1, 6, C2f, [256, True]] - [−1, 1, SPDConv, [512]] # 5-P4/16 - [−1, 6, C2f, [512, True]] - [−1, 1, SPDConv, [1024]] # 7-P5/32 - [−1, 3, C2f, [1024, True]] - [−1, 1, SegNext_Attention, []] # 9 - [−1, 1, SPPF, [1024, 5]] # 10

# YOLOv8.0-p2 head head: - [−1, 1, nn.Upsample, [None, 2, ‘nearest’]] - [[−1, 6], 1, Concat, [1]] # cat backbone P4 - [−1, 3, C2f, [512]] # 13

- [−1, 1, nn.Upsample, [None, 2, ‘nearest’]] - [[−1, 4], 1, Concat, [1]] # cat backbone P3 - [−1, 3, C2f, [256]] # 16 (P3/8-small)

- [−1, 1, nn.Upsample, [None, 2, ‘nearest’]] - [[−1, 2], 1, Concat, [1]] # cat backbone P2 - [−1, 1, EMA, []] - [−1, 3, C2f, [128]] # 20 (P2/4-xsmall) 20

- [−1, 1, SPDConv, [128]] - [[−1, 16], 1, Concat, [1]] # cat head P3 - [−1, 3, C2f, [256]] # 23 (P3/8-small) 23

- [−1, 1, SPDConv, [256]] - [[−1, 13], 1, Concat, [1]] # cat head P4 - [−1, 3, C2f, [512]] # 26 (P4/16-medium) 26

- [−1, 1, SPDConv, [512]] - [[−1, 10], 1, Concat, [1]] # cat head P5 - [−1, 3, C2f, [1024]] # 29 (P5/32-large) 29

- [[20, 23, 26, 29], 1, Detect_DyHead, [nc, 128, 1]] # Detect(P2, P3, P4, P5)
